# Mass Spectrometric Based Metabolomics of the Saudi Cultivar of Fenugreek (*Trigonella foenum-graecum* L.): A Combined GC-MS, Antimicrobial and Computational Approach

**DOI:** 10.3390/ph17121733

**Published:** 2024-12-21

**Authors:** Nujud A. M. Almuzaini, Abdel Moneim E. Sulieman, Naimah A. Alanazi, Riadh Badraoui, Emad M. Abdallah

**Affiliations:** 1 Department of Biology, College of Sciences, University of Hail, Hail P.O. Box 2440, Saudi Arabia; n.alenezy@uoh.edu.sa (N.A.A.); badraouir@yahoo.fr (R.B.); 2 Section of Histology-Cytology, Faculty of Medicine of Tunis, University of Tunis El Manar, La Rabta, Tunis 1007, Tunisia; 3 Department of Biology, College of Science, Qassim University, Buraydah 51452, Saudi Arabia; 140208@qu.edu.sa

**Keywords:** anti-bacterial agents, fenugreek, fungi, computational biology, in vitro study, chemical composition

## Abstract

**Background and Objective:** In Saudi Arabia, numerous plant species with promising medicinal properties are cultivated, widely traded, and commonly utilized in traditional medicine, including fenugreek (*Trigonella foenum-graecum*). This study aimed to comprehensively assess the phytochemical composition and antimicrobial potential of the Saudi cultivar of fenugreek using an integrative approach combining in vitro and in silico methodologies. **Methods:** A comprehensive investigation was conducted on the ethanol extract of fenugreek seeds, assessing its antibacterial, antifungal, properties. Computational modeling was employed to predict pharmacokinetic behavior and potential toxicity of the identified bioactive compounds. **Results:** Qalitative phytochemical analysis showed presence of alkaloids, tannins, saponins, glycosides, flavonoids, and steroids, while terpenoids were notably absent. GC-MS analysis of *Trigonella foenum-graecum* (fenugreek) seeds identified 25 bioactive compounds, with Ethyl methane sulfonate (12.41%) being the predominant component. Other key compounds included n-Hexadecanoic acid, 4-Butyl-2(4-nitrophenyl)-1,3-thiazole, and α-Tocopherol. In silico modeling of fenugreek phytochemicals supported their antibacterial, antioxidant, and neuroprotective potential, with compounds **21** and **24** showing strong binding to key targets like Tyrosyl-tRNA Synthetase (TyrRS) of *Staphylococcus aureus* (*S. aureus*), Aspartic proteinase from *Candida albicans* (*C. albicans*) and human peroxiredoxin 5. Pharmacokinetic analysis indicated good oral bioavailability, minimal CYP inhibition, and blood-brain barrier penetration, suggesting potential for treating neurodegenerative diseases. These bioactive compounds, including diosgenin and trigonelline, support fenugreek’s therapeutic promise and warrant further in vitro, in vivo, and clinical studies. **Conclusion:** The Saudi fenugreek cultivar is rich in bioactive compounds with good antibacterial potential. These findings establish a robust foundation for continued pharmacological research on the Saudi cultivar of *T. foenum-graecum*, highlighting its potential as a rich source of bioactive compounds with significant medicinal value.

## 1. Introduction

Medicinal plants have been exploited as a source of medicine across almost all societies from antiquity, with fossil evidence dating back 60,000 years in Iraq, when ancient people employed medicinal plants for therapeutic purposes [1,2]. The growing interest in medicinal plants as complementary health care options has been driven by the escalating costs of modern medicine and concerns over its side effects. Traditional medicinal practices are widely prevalent in Eastern countries such as China, India, Pakistan, Sri Lanka, Thailand, and Japan. In China, herbal treatments account for over 40% of total medical consumption [3]. The growing interest in medicinal plants has led to a significant rise in both investment in scientific research and the global demand for these natural remedies. In 2020, the global market for medicinal plant-based products reached a turnover of $100.9 billion, with projections indicating continued growth in the coming years [4]. According to the World Health Organization (WHO), “herbal medicines” or medicinal plants are materials or products derived from plants that offer therapeutic or other health benefits, containing raw or processed components from one or multiple plants [5]. Moreover, concerns are increasing regarding a potential regression to a pre-antibiotic era, characterized by the prevalence of widespread outbreaks of severe bacterial infections. The studies highlight the exacerbation of epidemics by environmental stressors, including climate change, resource depletion, and detrimental human activities such as warfare, conflict, antibiotic overuse, and pollution [6]. Medicinal plants hold significant potential as sources of antimicrobial agents due to their rich phytochemical profiles. Many plant-derived compounds exhibit antibacterial, and antifungal properties, offering alternative solutions to combat drug-resistant pathogens. Ongoing research is crucial to harness their full therapeutic potential [7,8,9].

Fenugreek (*Trigonella foenum-graecum* L.) is a famous medicinal plant. It is a versatile herb with a long history of culinary, medicinal, and therapeutic applications, particularly in Western Asia and the Mediterranean region. Fenugreek seed oil is notable for its high content of unsaturated fatty acids, such as oleic acid and linoleic acid, along with a rich profile of antioxidants. Additionally, fenugreek seeds are packed with essential nutrients, such as vitamins A, C, B6 (including thiamine, pyridoxine, folic acid, and niacin), and minerals like magnesium, iron, and manganese. Additionally, they are a significant source of soluble fiber and various phytonutrients. Its notable antibacterial properties have shown efficacy against various bacterial and fungal strains [10,11]. Fenugreek exhibits a broad range of biological activities, such as hypocholesterolemic, antibacterial, antiparasitic, anticancer, and antidiabetic effects [12]. Aasim et al. and Rashid et al. [13,14] have highlighted the significant phytochemical properties of fenugreek, leading to its diverse applications in culinary, nutraceutical, and medicinal fields. As noted by Ruwali et al. [15], fenugreek’s therapeutic potential supports its use in nutraceuticals, while its unique flavor profile makes it a popular culinary ingredient. Various extraction techniques have been employed to enhance the concentration of bioactive compounds, optimizing its application in the food industry for flavor enhancement. Fenugreek also demonstrates hepatoprotective, anticancer, lactogenic, and hypoglycemic effects, attributed to its rich composition of polyphenols, galactomannans, alkaloids, and saponins [13,14,15,16].

Fenugreek demonstrates significant antibacterial properties attributed to its phytochemicals, which function via many pathways [17]. Research demonstrates its effectiveness against multiple bacteria, including *Streptococcus mutans*, *Lactobacillus*, *Enterococcus faecalis*, and *Candida albicans*. Fenugreek gel at 100 µg/mL exhibited a notable zone of inhibition (5.39 ± 0.05 mm) in contrast to doxycycline (1.1 ± 0.08 mm) against oral microorganisms [18,19]. Sprout extracts demonstrated efficacy against *Escherichia coli* (*E. coli*) and *Klebsiella pneumoniae* (*K. pneumoniae*) Nonetheless, crude extracts exhibited restricted efficacy against Gram-negative bacteria, indicating that activity is associated with particular chemical constituents [18,19]. Ethanolic extracts demonstrated superior efficacy compared to aqueous extracts, exhibiting enhanced antibacterial activity, extended shelf life, and reduced vulnerability to contamination [18,19]. These findings underscore the potential of fenugreek bioactives as antibacterial agents, particularly in ethanolic formulations.

The Arabian Peninsula is mostly a desert area characterized by little biodiversity. Nonetheless, the Kingdom of Saudi Arabia (KSA) has a diverse array of plant species, including several edible and medicinal plants, along with varied trees, shrubs, and herbs. Notwithstanding this abundance, the medicinal potential of these plants remains mostly unexamined by the scientific community [20]. In Saudi Arabia, interest in agriculture is steadily increasing, with certain regions recognized for their thriving agricultural activities. Key areas include the central regions, encompassing Hail, Qassim, Riyadh, and Wadi ad-Dawasir, which are known for their agricultural productivity [21].

Studies on the biological properties of the Saudi Cultivar of Fenugreek cultivated in Saudi Arabia are scarce. It is well known that, geographic variation significantly influences plant characteristics, including morphology, physiology, and phytochemical composition. Differences in climate, soil type, altitude, and environmental stressors across regions can lead to the development of unique traits and secondary metabolites in plants. This variation impacts their medicinal, nutritional, and ecological properties [22,23,24]. Moreover, significant differences in the chemical and nutritional profiles of *T. foenum-graecum* seeds have been observed among those collected from various countries, such as India, Saudi Arabia, Turkey, and Yemen [25]. Accordingly, this study aimed to comprehensively evaluate the phytochemical profile and antimicrobial potential of the Saudi cultivar of *Trigonella foenum-graecum* (fenugreek) through a combination of in vitro and in silico approaches by targeting Tyrosyl-tRNA Synthetase (TyrRS) from *Staphylococcus aureus (S. aureus)*, aspartic proteinase from *Candida albicans* (*C. albicans)*, and human peroxiredoxin 5.

## 2. Results

### 2.1. Physico-Chemical Properties of Fenugreek Seed Oil

Table 1 presents the physicochemical properties of fenugreek seed oil. The recorded values include an acid value of 4.75 mg KOH/g, a saponification value of 195 mg KOH/g, an ester value of 190.25, free fatty acids at 2.38 mg/100 g of oil, and a refractive index of 1.4640 at 37 °C. Notably, the strong odor characteristic of fenugreek seed oil diminishes upon consumption.

### 2.2. Qulalitative Phytochemical Properties of Fenugreek Seed

Table 2 highlights the primary classes of compounds identified in *T. foenum-graecum* (fenugreek) seeds. The analysis reveals that fenugreek is abundant in alkaloids, tannins, saponins, glycosides, flavonoids, and steroids, while terpenoids were notably absent.

### 2.3. Gas Chromatography/Mass Spectrometry (GC-MS) Results

**Figure 1:**
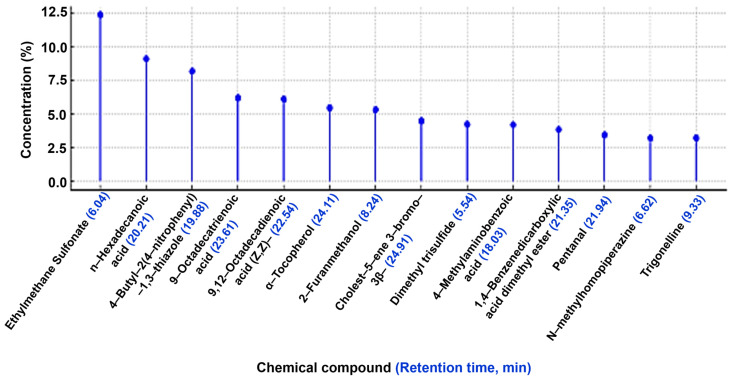
Chromatogram of the ethanol extract of fenugreek seeds.

Table 3 and Figure 1 summarize the compounds identified in the ethanol extract of fenugreek seeds, along with their retention times, molecular formulas, and relative concentrations. A total of 25 compounds were detected, with Ethyl methane sulfonate (12.41%) emerging as the predominant component. This was followed by n-Hexadecanoic acid (9.12%), 4-Butyl-2(4-nitrophenyl)-1,3-thiazole (8.21%), 9-Octadecatrienoic acid (6.23%), 9,12-Octadecadienoic acid (Z,Z) (6.12%), α-Tocopherol (5.46%), 2-Furanmethanol (5.32%), Cholest-5-ene 3-bromo- (4.51%), Dimethyl trisulfide (4.23%), Methylaminobenzoic acid (4.21%), 1,4-Benzene dicarboxylic acid dimethyl ester (3.86%), Pentanal (3.45%), N-Methylhomopiperazine (3.22%), and Trigonelline (3.21%).

### 2.4. Antibacterial Properties of Fenugreek Oils

The antimicrobial activity of the ethanol extract of *T. foenum-graecum* (fenugreek) seeds is detailed in Table 4. The extract demonstrated efficacy against the tested microorganisms, including *S. aureus*, two gram-negative *E. coli* strains, and *Sal typhimurium*, at concentrations of 1, 2, and 3 mg/disc. At the maximum concentration evaluated (3 mg/disc), the extract exhibited a statistically significant inhibitory effect (*p* < 0.05) in comparison to the reference antibiotic, ampicillin (10 µg/disc). This suggests that the extract demonstrates significant antibacterial action, albeit it necessitates a greater concentration than ampicillin to attain a similar effect. At this concentration, the ethanol extract produced an inhibition zone of 15.00 ± 1.5 mm against *E. coli* and 16.11 ± 0.10 mm against *Sal. typhimurium*. Notably, the extract exhibited a more pronounced antibacterial effect against *E. coli*, with inhibition zones of 12.56 ± 0.12 mm, 13.44 ± 0.25 mm, and 16.11 ± 0.10 mm at concentrations of 1, 2, and 3 mg/disc, respectively.

The data also highlight the extract’s significant antifungal activity against *A. flavus*. At 1 mg/disc, the inhibition zone measured 14.11 ± 0.12 mm, increasing to 16.87 ± 0.32 mm at 3 mg/disc. The results indicate that the fenugreek extract exhibits stronger antifungal activity compared to antibacterial effects, and its inhibitory potential increases with higher concentrations of the extract.

The fenugreek-identified phytochemicals had different affinities to TyrRS from *S. aureus* (Protein Data Bank ID: 1JIJ), aspartic proteinase from *C. albicans* (Protein Data Bank ID: 2QZW) receptors, and human peroxiredoxin 5 (PRDX5, Protein Data Bank ID: 1H2D) (Table 5). Recently, it has been reported that binding affinities depend mainly on the 3D chemical structure of the ligands and their structural geometry [26,27,28]. In the current work, the 25 identified compounds of Fenugreek had negative binding affinities, which support their biological effects. The best binding affinities reached −9.4 kcal/mol for 1JIJ, −8.6 kcal/mol for 2QZW, and −8.3 kcal/mol for 1H2D. These scores concerned mainly compounds no. **21** and **24**. Compound no. **21** had the highest affinity and established good molecular interactions with the 1JIJ (Table 5 and Table 6).

**Figure 2:**
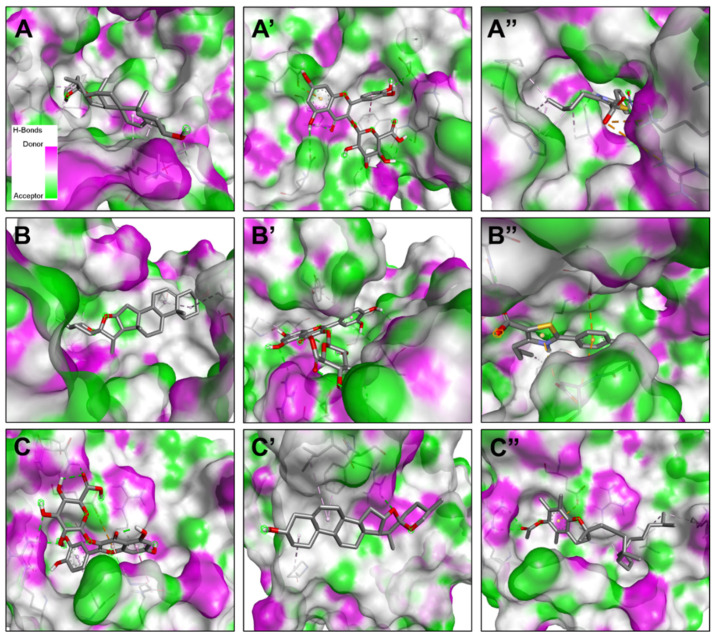
Tridimensional illustrations of the 3 targeted receptors 1JIJ (**A**–**A″**), 2QZW (**B**–**B″**) and 1H2D (**C**–**C″**) with the three predicted best compounds identified in the fenugreek seeds. 1JIJ complexed with compounds no. **21** (**A**), **24** (**A′**), and **15** (**A″**). 2QZW complexed with compounds no. **21** (**B**), **24** (**B′**), and **15** (**B″**). 1HD2 complexed with compounds no. **24** (**C**), **21** (**C′**), and **23** (**C″**).

**Figure 3:**
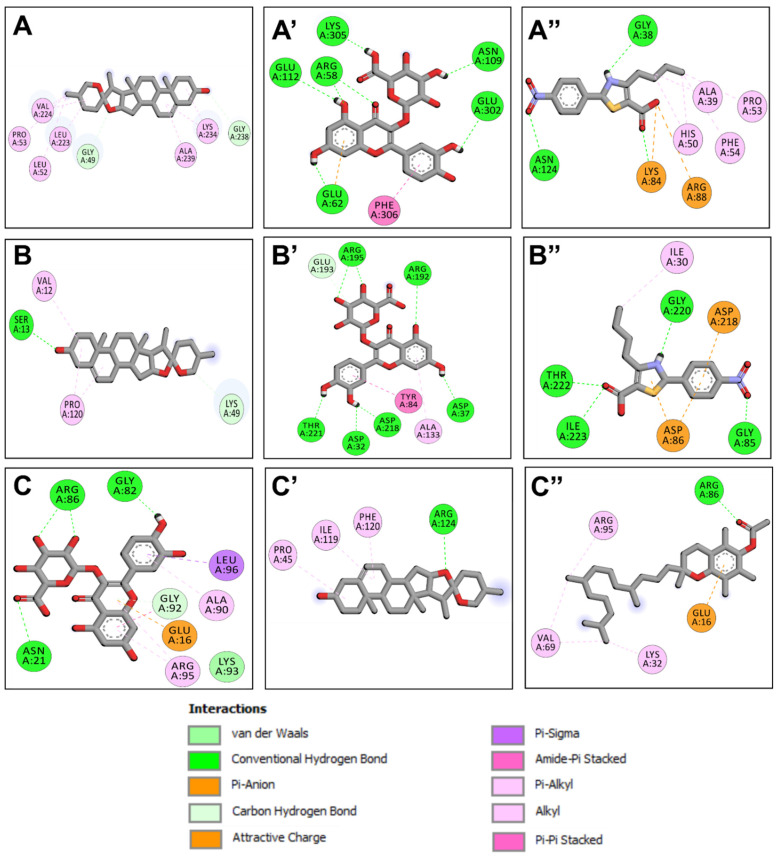
Illustration of the 2D diagrams of interactions of the 3 targeted receptors 1JIJ (**A**–**A″**), 2QZW (**B**–**B″**) and 1H2D (**C**–**C″**) with the three predicted best compounds identified in the fenugreek seeds. 1JIJ complexed with compounds no. **21** (**A**), **24** (**A′**), and **15** (**A″**). 2QZW complexed with compounds no. **21** (**B**), **24** (**B′**), and **15** (**B″**). 1HD2 complexed with compounds no. **24** (**C**), **21** (**C′**), and **23** (**C″**).

**Figure 4:**
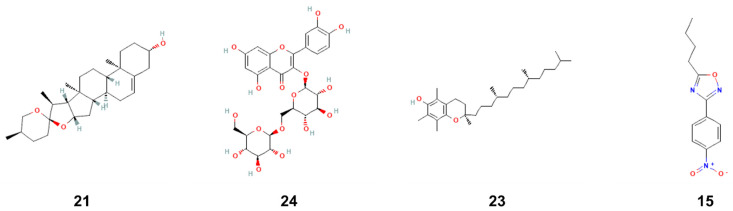
Chemical structure of the best compounds (**15**, **21**, **23** and **24**) from the docking analysis.

The molecular interactions included two carbon H-bonds associated with a network of hydrophobic bonds that contribute to the stability of the complex [27,28,29] (Figure 2 and Figure 3). These interactions concerned several key residues. It has been found that it included eight different residues: once for each of Gly49, Gly238, Leu223, Ala239, Leu52, and Pro53, and twice for each of Val224 and Lys234 (Table 5 and Figure 3). Compound no. **24** established the highest number of conventional H-bonds with both 1JIJ and 2QZW. Compounds no. **21** and **24** were predicted as the most active for the three targeted receptors and showed deep embedding. Previously, it has been reported that tight embedding (<2.5 Å), as those outlined in our study, is usually associated with potential biological effects, including anti-inflammatory, antiproliferative, antimicrobial, and antioxidant effects [28,29]. Overall, the in-silico modeling approach outlines that both antimicrobial and antioxidant effects of fenugreek compounds are thermodynamically possible. These biological processes were actually reported by the current study through in vitro tests. Our results support the beneficial, promising impact and health promotion of natural-derived compounds, phytotherapy, and medicinal plants, including *Trigonella foenum-gracum* L. [29,30]. The chemical structure of compound no. **15**, **21**, **23**, and **24** are shown (Figure 4).

**Figure 5:**
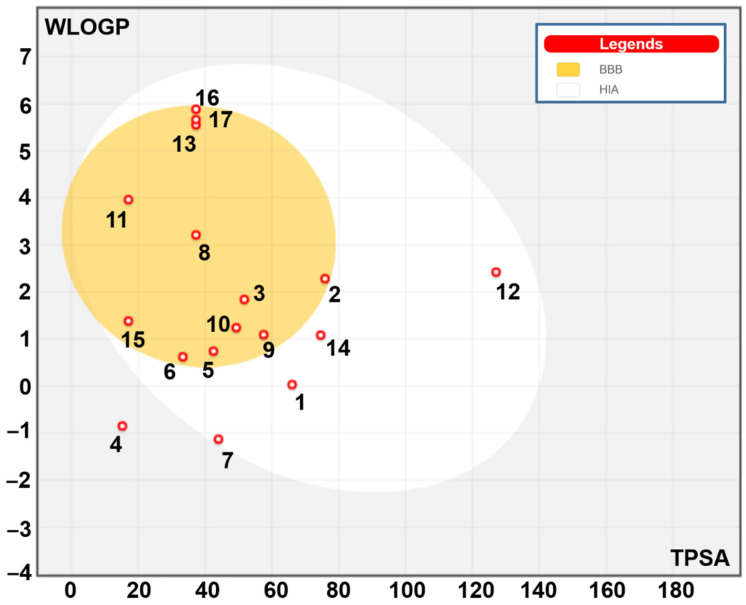
Boiled-egg model of the identified phytochemicals. The yellow and white areas correspond to the BBB (for blood-brain-barrier) permeation and GI (for gastro-intestinal) absorption, respectively.

Table 7 exhibited the pharmacokinetics, drug-likeness, and medicinal chemistry of the fenugreek-identified compounds based on their ADME/Tox (for absorption, distribution, metabolism, excretion, and toxicity) properties. The pharmacokinetic analyses revealed acceptable drug-likeness and medicinal chemical properties for most identified compounds. Interestingly, most of the identified compounds did not inhibit the five assessed cytochrome P450 (CYPs) isoforms (CYP1A2, CYP2C19, CYP2C9, CYP2D6, and CYP3A4) and possessed good oral bioavailability. Furthermore, most of the fenugreek compounds stand on white and yellow areas of the mapped boiled egg model (Figure 5), which indicates that these phytochemicals are predicted to be passively absorbed by the G.I. tract and passively permeate the BBB, respectively.

## 3. Discussion

### 3.1. Chemical Profile of Fenugreek Seeds

The phytochemical analysis of *Trigonella foenum-graecum* (fenugreek) seeds revealed a diverse array of bioactive compounds, notably the absence of terpenoids. This finding aligns with prior research by Mahmood and Yahya [31] and Shehab et al. [18,32], which documented the presence of several significant phytochemical groups in fenugreek, including alkaloids, glycosides, tannins, saponins, steroids, and flavonoids. Moreover, fenugreek seeds are known to contain roughly 35% alkaloids, mostly trigonelline. Furthermore, fenugreek seeds contain more than 10 mg of flavonoids per gram, in addition to trace amounts of both volatile and fixed oils [33]. The bitter taste and unique aroma of fenugreek seeds are mainly due to the alkaloid and volatile substances contained inside. Furthermore, polyphenolic chemicals such as rhaponticin and isovitexin are regarded as the principal bioactive elements that provide the therapeutic effects of fenugreek seeds [34]. These compounds contribute to fenugreek’s well-documented biological activities such as antioxidant, antimicrobial, and anti-diabetic activity [35]. Unlike many other therapeutic plants that are rich in terpenoids, fenugreek’s lack of these compounds suggests a distinctive specialization in its bioactive composition. Flavonoids and alkaloids, both of which are abundant in fenugreek seeds, may play pivotal roles in its biological activities. Flavonoids, known for their anti-inflammatory and antioxidant properties, help alleviate oxidative stress and protect cellular structures [29,36,37], while alkaloids exhibit antibacterial properties by disrupting microbial cell walls or inhibiting protein synthesis [38].

### 3.2. GC-MS Profiling of Fenugreek Seed Extracts

The GC-MS analysis of fenugreek seed extracts identified twenty-five distinct chemical compounds, spanning a wide range of functional groups. The predominant components include six acids, one ester, six phenols, and one ether, with phenolic compounds being the major contributors to the plant’s bioactivity. These phenolic compounds are known for their antioxidant, antibacterial, anti-inflammatory, and antiproliferative properties [27,29,39]. Among the most notable compounds identified were Trigonelline (retention time 9.33 min) and Diosgenin (retention time 23.13 min), which are key bioactive compounds widely associated with Fenugreek [40]. Trigonelline is a quaternary alkaloid that has demonstrated various pharmacological activities, including anti-inflammatory, neuroprotective, and antidiabetic effects [41]. Its presence in the extract supports the potential therapeutic applications of Fenugreek in managing diabetes and other inflammatory conditions. Diosgenin, a steroidal saponin, is recognized for its estrogenic, anti-inflammatory, and anticancer properties, further highlighting the significance of this plant as a source of bioactive phytochemicals with broad therapeutic potential [42,43]. In addition to these key compounds, several other metabolites were identified, including 4-Hydroxybenzoic acid, a phenolic compound with known antioxidant and antibacterial properties, and this compound was found to be greatly affected by geographical conditions as reported with Egyptian Fenugreek [44]. α-Tocopherol, serves as a potent antioxidant and contributes to the overall health benefits of the plant. These compounds reinforce the antioxidative and antimicrobial potential of Fenugreek, making it a valuable candidate for nutraceutical and pharmaceutical applications. The presence of Dimethyl trisulfide and Ethylmethane sulfonate, both sulfur-containing compounds, suggests the possibility of additional therapeutic activities related to their antimicrobial and anti-inflammatory effects. Sulfur compounds have been reported to exert beneficial effects against a variety of pathogens, further supporting the broad-spectrum bioactivity of Fenugreek. Notably, Dodecamethylcyclohexasiloxane (14.2 min), a silicone-based compound, was identified in the extract, which is not typically associated with Fenugreek and may suggest environmental or processing factors influencing the chemical composition of the sample. However, the predominant compounds that define Fenugreek’s bioactive profile—such as Trigonelline and Diosgenin—remain consistent with its established therapeutic properties. The identification of β-Estradiol-3-methyl ether raises interesting questions regarding the estrogenic potential of Fenugreek. Although estrogenic compounds have been previously isolated from Fenugreek (e.g., phytoestrogens), further studies are required to determine whether this compound contributes to the plant’s reputed effects on hormone regulation and reproductive health. Further GC-MS associated investigations on the relationships among MS measurable variables, molecular properties, and molecular structural parameters from the perspective of chemometrics as recently reported by Upadyshev et al. [45] would have additional value to the phytochemical analyses of Fenugreeks.

### 3.3. Antimicrobial Activity and Mechanisms

The antibacterial efficacy of fenugreek seed extract demonstrated a marked difference in its activity against Gram-positive and Gram-negative bacteria, which can be attributed to their distinct cell wall structures [46]. The outer membrane of Gram-negative bacteria, enriched with lipopolysaccharides (LPS), acts as a formidable barrier to many plant-derived antimicrobial agents, including hydrophobic compounds [47]. In contrast, the absence of such a protective barrier in Gram-positive bacteria allows fenugreek’s bioactive compounds to exert a more potent effect. This suggests that fenugreek seed extract may be especially effective against infections caused by *Bacillus subtilis* [22]. The antimicrobial action likely occurs through the disruption of bacterial cell membranes and interference with essential cellular functions [37]. Saponins and alkaloids, abundant in fenugreek, have been shown to modify membrane permeability, potentially leading to cell lysis and the release of intracellular contents [48]. This mechanism reinforces the potential of fenugreek as a natural alternative to synthetic antibiotics, especially given the escalating concerns surrounding antibiotic resistance. The fenugreek extract exhibited dose-dependent inhibition against *S. aureus*, with inhibition zones ranging from 9.12 ± 1.15 mm at 1 mg/disc to 15.02 ± 0.50 mm at 3 mg/disc. These values, although significant, were lower than the standard antibiotic ampicillin (25.25 ± 1.12 mm), suggesting moderate efficacy against gram-positive bacteria. Our study agrees with Idris et al. [49] who cited that fenugreek good antibacterial activity against different bacterial pathogens including *S. aureus* and methicillin-resistant which causes several difficult-to-treat infections in humans. In outs study, the extract showed also good activity against *E. coli*, with inhibition zones improving from 12.56 ± 0.12 mm at 1 mg/disc to 16.11 ± 0.10 mm at 3 mg/disc. Interestingly, the extract at higher concentrations outperformed the standard ampicillin (11.22 ± 0.33 mm), highlighting its strong antibacterial properties against this gram-negative strain. Here, our results were in clear contrast to one previous study which showed that the aqueous extract of fenugreek has no activity against *E. coli* and chloroform, acetone, ethanol, and methanol extracts showed weak to moderate activity against *E. coli* [50]. This confirms that the biological activities of the fenugreek greatly varies between cultivars. Another study stated that the fenugreek seed extract at a concentration of 40 mg/mL exhibited the highest sensitivity, effectively inhibiting the growth of extended-spectrum beta-lactamase (ESBL)-producing *E. coli* [51]. A similar dose-dependent response was observed against *Sal. typhimurium*, with inhibition zones increasing from 8.72 ± 0.30 mm to 15.24 ± 0.10 mm. The extract at its highest concentration outperformed the standard antibiotic (7.42 ± 0.27 mm), indicating its potential against this pathogen. It was mentioned that, both aqueous and methanol extracts of fenugreek seeds demonstrate notable antibacterial activity against various bacterial strains, including *Pseudomonas* spp., *E. coli*, *Shigella dysenteriae*, and *Sal. typhimurium* [52]. Moreover, the extract showed significant antifungal activity, with inhibition zones ranging from 14.11 ± 0.12 mm to 16.87 ± 0.32 mm. Although the activity was slightly lower than amphotericin B (21.65 ± 1.22 mm), the extract demonstrated promise as an antifungal agent. It was published that a defensin-like antifungal peptide has been isolated from fenugreek seeds, demonstrating effective inhibition of fungal growth, including species such as *Fusarium oxysporum*, *Fusarium solani*, and *Rhizoctonia solani* [18]. This highlights its potential as a natural antifungal agent for agricultural and medicinal applications.

### 3.4. In Silico/Computational Modeling of Fenugreek

In silico and computational modeling of fenugreek phytochemicals revealed their biological and pharmacokinetic characteristics. The docking investigations showed that the discovered compounds have substantial binding affinities to major molecular targets, including TyrRS from *S. aureus*, aspartic proteinase from *C. albicans*, and human peroxiredoxin 5. These antibacterial and antioxidant molecular targets are important for understanding fenugreek’s pharmacological efficacy.

The docking results showed that all the 25 fenugreek compounds had negative binding affinities. The lowest binding affinity scores were −9.4 kcal/mol for 1JIJ, −8.6 for 2QZW, and −8.3 for 1H2D, particularly with compounds no. **21** and **24**. These binding affinities indicate that phytochemicals attached to the targeted receptors are thermodynamically possible and stable, indicating their potential biological activity. Recently, it has been reported that binding affinities depend mainly on the 3D chemical structure of the ligands and their structural geometry [26,27,28].

Compound no. **21** possessed the highest binding affinity for 1JIJ and established acceptable molecular interactions, which suggest that it may inhibit TyrRS, a bacterial protein production enzyme. It shows promising antibacterial activity, especially against *S. aureus.* The interactions of compound no. **24** with 1JIJ and 2QZW reveal a broad-spectrum antibacterial activity against bacterial and fungal infections. The ligand-receptor complexes were stable due to hydrogen bonding and hydrophobic interactions. Key interacting residues included Gly49, Val224, Lys234, and Leu223. 

The deep embedding of compounds no. **21** and **24** (<2.5 Å) in binding sites is significant, as this closeness is often linked to powerful biological effects such as antibacterial, antioxidant, anti-inflammatory, and antiproliferative properties [29,36]. Previous investigations have shown that tight binding contacts increase natural chemicals’ target inhibition.

### 3.5. Pharmacokinetics, Drug-likeness, ADME/Tox

The ADME/Tox (absorption, distribution, metabolism, excretion, and toxicity) profiles of fenugreek phytochemicals revealed their drug-likeness. Prediction of these parameters is of key importance to avoid any drug failure at advanced stages [27,30,36]. Most substances demonstrated good oral bioavailability and low toxicity, according to the assessments. One important finding was that most drugs did not inhibit the five main cytochrome P450 (CYP) isoforms. CYP enzymes are essential for medication, metabolism and inhibiting them can produce side effects [27,36]. The fact that most fenugreek compounds did not inhibit these isoforms suggests fewer drug-drug interactions and improved therapeutic safety.

Fenugreek compounds’ pharmacokinetic potential was supported by the boiled egg model. The model mapped substances in the white and yellow patches, which represent passive G.I. absorption and BBB permeability.

### 3.6. Future Directions and Implications

Molecular docking, pharmacokinetics, and ADME/Tox investigations reveal fenugreek’s potential as a natural medicinal extract. Its significant binding affinities and molecular interactions with microbial targets support the antibacterial capabilities, and its good pharmacokinetics imply a safe and effective oral therapy [28,29]. Several fenugreek chemicals can penetrate the BBB, suggesting they could treat neuroprotective or neurodegenerative illnesses [27,36,53].

Fenugreek compounds **21** and **24**’s antimicrobial and antioxidant properties may lead to new bacterial and fungal infection treatments. Clinical trials are needed to evaluate the safety and efficacy of fenugreek compounds in humans, particularly for long-term usage and drug interactions.

The in-silico investigation shows that fenugreek phytochemicals have antibacterial, antioxidant, and therapeutic potential [36]. These natural chemicals’ bioavailability and biological efficiency are enhanced by their binding affinities, molecular interactions, and pharmacokinetics.

### 3.7. Justification for Pharmaceutical Applications

The potential medicinal uses of fenugreek seeds are supported by the presence of several identifiable chemicals in them. The bioactive steroidal saponin diosgenin has been the subject of substantial research due to its potential anti-inflammatory, cholesterol-lowering, and anticancer effects. Its inclusion in fenugreek seeds boosts the extract’s medicinal potential, especially in pharmacological treatments for metabolic and inflammatory diseases [54].

Another important component with pharmacological promise is trigonelline, an alkaloid present in fenugreek seeds. An important component in herbal therapy has been demonstrated to have neuroprotective, hypoglycemic, and anticancer properties. One of the most significant components in fenugreek, trigonelline, shows that the seed could heal a variety of diseases and cancers [55].

In the seeds, you can find complexes of flavonoids including quercetin and apigenin, which are well-known for their powerful antioxidant capabilities. By preventing oxidative damage to cells, these chemicals neutralize free radicals and lower the risk of chronic diseases including cancer, heart disease, and neurological problems. Fenugreek has anti-inflammatory and antioxidant properties due to its flavonoid and phenolic acid combinations, such as 4-hydroxybenzoic acid.

## 4. Materials and Methods

### 4.1. Plant Materials

Fenugreek seed samples cultivated in the Hail region were procured from an authorized store and authenticated by a botanist in the Department of Biology, College of Sciences, University of Hail, Saudi Arabia, in 2023. The firm, tiny, golden-yellow seeds of Fenugreek are bitter and have a distinctive smell. Mature seeds are employed to test antimicrobial activity since they are completely formed, have higher secondary metabolite concentrations, and contain medicinal and therapeutic qualities. Younger seeds may not contain all bioactive chemicals, whereas mature seeds have more antibacterial properties. The seeds were thoroughly rinsed with distilled water, air-dried in the shade, and ground into a fine powder using a pestle and mortar. The powdered seeds were stored in well-sealed glass bottles in a dark place to preserve their integrity until further use.

### 4.2. Proximate Analysis

Proximate analysis was performed to evaluate the essential components of dried fenugreek seed samples, including moisture, protein, fiber, ash, and fat contents, following previously established procedures [56]. Carbohydrate content was calculated by subtracting the total percentages of moisture, protein, fiber, ash, and fat from 100. All analyses were conducted in triplicates, and the mean values were determined to ensure accuracy.

### 4.3. Extraction and Physicochemical Properties

Fenugreek seed oil was extracted using n-hexane via the Soxhlet apparatus [57]. The extraction process was repeated multiple times to obtain sufficient oil for analysis. Physicochemical properties of the extracted oil were evaluated according to the Standard Methods for Examination of Fats, Oils, and Derivatives [58], measuring parameters such as acid value, saponification number (mg KOH/g oil), ester value, free fatty acid content (as oleic acid per 100 g oil), and refractive index at 37 °C. Essential oils were extracted separately using hydro-distillation with a Clevenger-type apparatus for 3 h, then dried with anhydrous Na₂SO₄ and stored in sealed vials at 4 °C in the dark. The essential oil yield was determined gravimetrically based on the dry weight of the seeds [59].

### 4.4. Preparation of Microbial Specimens

The efficacy of fenugreek oil was evaluated against a single strain of gram-positive bacterium, *Staphylococcus aureus* (*S. aureus*), and two strains of gram-negative bacteria, *Escherichia coli* (*E. coli*) and *Salmonella enterica* subsp. *enterica* serovar Typhimurium (*S. typhimurium*), and one strain of mold, *Aspergillus flavus (A. flavus).* The bacteria used in the experiment were acquired from the Food Microbiology Laboratory at our labs.

### 4.5. Cultivation of the Test Organisms

The surface viable counting technique determined the average concentration of viable organisms per milliliter (mL) in the stock suspensions. For each trial, a new stock suspension was prepared, ensuring that the experimental settings remained consistent. This allowed us to obtain suspensions with remarkably similar viable counts. The *A. flavus* fungal culture was cultivated on Saboraud dextrose agar and incubated at 25 °C for four days. The mycelium was collected and rinsed with sterile isotonic saline solution, then resuspended in 100 mL of sterile isotonic saline solution. The resulting suspension was refrigerated for future use.

### 4.6. Testing of Plant Extract for Antimicrobial Activities

The antibacterial and antifungal properties of the plant extract were assessed using the agar well diffusion method, as demonstrated by Daoud et al. [60]. A volume of 1 mL of a recently cultivated bacterial or fungal culture was transferred using a pipette and placed at the center of a sterile Petri dish. Subsequently, the inoculum was combined with molten cooled Muller Hinton agar (MHA) for bacteria strains or Potato dextrose agar (PDA) for fungi in a Petri plate, ensuring thorough mixing. After the agar plates containing inoculums solidified, wells were created using a sterile cork borer with a diameter of 6 mm. The plates were incubated at 37 °C for 24 to 48 h. The fenugreek seed extract was applied on sterile discs using three different dosages (1 mg, 2 mg, and 3 mg per disc). Every concentration was evaluated three times against the target species. The growth inhibition diameter zones were recorded together with the average and the mean values after incubation. Ampicillin and amphotericin B were used as reference molecules.

### 4.7. Phytochemical Profile

Following Raaman [61] and Banso and Adeyemo [46] procedures, phenols, tannins, alkaloids, flavonoids, terpenoids, sterols, cardiac glycosides, and saponins were found in fenugreek seeds.

Phenols: 10 mL ethanol extract was added to 3 drops of 5% FeCl_3_. A bluish-black color implies phenolic chemicals.

Alkaloids: In Dragendorff’s test, 500 µL of Dragendorff’s reagent was added to 5 mL of ethanol extract along the test tube side after adding 2 mL MeOH and 2 mL 1% HCl. Orange or orange reddish-brown precipitate indicated a positive result. The extract was tested with two drops of Mayer’s reagent in 1 mL. White or creamy precipitate indicates alkaloids.

Flavonoids: Adding 2 mL NaOH 2% to 5 mL extract made it intensely yellow. The hue disappears when diluted HCl is added, indicating flavonoids.

Salkowski-tested terpenes: Mixing 5 mL extract with 2 mL chloroform. Then 3 mL conc. H_2_SO_4_ was added. A reddish-brown color implies terpenoids.

Steroids: 5 mL extract with 2 mL H_2_SO_4_ received 2 mL glacial acetic anhydride. Color changes from violet to blue or green suggest steroids.

Keller-Kiliani glycosides: Three drops of 5% FeCl_3_ and 1 mL of glacial acetic acid were added to 2.5 mL of extract. On the side of the test tube was put 0.5 mL of concentrated H_2_SO_4_. Cards containing cardiac glycosides will be colored green or blue.

Saponins content: We added 10 mL distilled water to 3 g seed powder. Five minutes were spent shaking the solution. Stable foam indicates saponins.

Tannins: 3 drops of 5% FeCl_3_ solution were added to 2 mL of diluted extract. The green, black, or blue color indicated tannins.

### 4.8. GC–MS Analysis 

The GC–MS study used a Perkin Elmer Clarus 600 GC System with an Rtx 5MS capillary column (30 m 0.25 mm i.d. 0.25 m film thickness; max. temp. 350 °C) and Clarus 600C MS. The carrier gas was ultra-high-purity helium (99.9999%) flowing at 1.0 mL/min. Ion source, transfer line, and injector temperatures were 280, 270, and 270 °C. This gas ionized at 70 eV. The EM voltage was estimated using autotune. Every data came from full-scan mass spectra between 40 and 550 amu. Analysis conditions: The split ratio of 1 L injected sample was 10:1. The oven was programmed to maintain 280 °C for 25 min at 80 °C each minute from 60 °C. Conditions for G.C.–M.S. leaf oil analysis: As noted, GC–MS detected FAME molecules. The helium flow was 0.7 mL/min. The ion source, transfer, and injector were heated to 250, 250, and 220 °C. After starting at 50 °C (kept for 1 min), the oven was heated to 250 °C at 40 °C per min. All data was collected by obtaining full-scan mass spectra from 35 to 500 amu. Spectra were compared to mass spectral libraries to identify compounds [32]. We used manufacturing conditions to determine the system’s calibration and minimal detection limits. The equation is available elsewhere [33].

### 4.9. In-Silico Study

The antimicrobial and antioxidant activities of the fenugreek seed oil were also assessed using in silico modeling and interaction assays. For this purpose, the TyrRS from *S. aureus* (1JIJ), the aspartic proteinase from *C. albicans* (2QZW), and human peroxiredoxin 5 (PRDX5, 1H2D) have been retrieved from RCSB data bank. Then, the active sites of these receptors were targeted to study the antibacterial, antifungal, and antioxidant effects, respectively. ChemDraw was used to draw the chemical structures of the fenugreek seed oil if the compound did not exist on the PubChem website. The docking approach was carried out based on the CHARMm force field as previously published [26,29,53] following the preparation of both ligands and receptors by removing water molecules and supplementing both polar hydrogens and Kollman charges. The assessment of binding scores bond categories were further analyzed, for the best three compounds, as previously reported [26,27,29]. 1JIJ, 2QZW, and 1H2D have been selected as they are commonly associated with the pathogenesis of infectious diseases, particularly from *S. aureus* and *C. albicans* and pro-antioxidant pathways [26,29,30,36].

Bioavailability and pharmacokinetic properties of the fenugreek-identified phytochemicals have also been studied as previously described [28,29]. The computational assessment of these parameters was based on the ADME/Tox measurements (for absorption, distribution, metabolism, excretion, and toxicity) [30,36].

### 4.10. Statistical Analysis

The statistical analyses were conducted using the SPSS software package (version 18). The comparisons were executed through one-way ANOVA, followed by a Tukey post hoc test. A level of *p* < 0.05 was considered statistically significant. The results are reported as mean ± standard deviation (SD).

## 5. Conclusions

Research using gas chromatography-mass spectrometry on *Trigonella foenum-graecum* has confirmed its anti-inflammatory, antioxidant, and antibacterial claims by revealing a number of bioactive chemicals, including trigonelline, diosgenin, and 4-hydroxybenzoic acid. The plant’s adaptability in medicine and its potential for novel applications are highlighted by the discovery of new compounds. The results were confirmed by computational analyses, which showed that important compounds, especially **21** and **24**, have strong binding affinities to key targets such as TyrRS (*S. aureus*), aspartic proteinase (*C. albicans)*, and human peroxiredoxin 5. Promising antibacterial, antioxidant, and neuro-protective potential is suggested by favorable pharma-cokinetics, which include excellent oral bioavailability, low CYP isoform inhibition, and blood-brain barrier permeability. Among plant-based antimicrobials, fenugreek shows promise due to its dose-dependent antibacterial effect, which is especially noticeable against *Sal. typhimurium* and *E. coli*. It needs further modification to increase its potency, nevertheless, because it is only moderately efficient against *S. aureus* and *A. flavus*. The bioactive components of fenugreek should be isolated and identified in future efforts. Their synergistic effects should be studied, and extraction and formulation methods should be fine-tuned. Its pharmacological properties must be confirmed by in vivo and clinical studies, and its ability to cross the blood-brain barrier calls for study into neurotherapeutic applications. Based on these findings, *T. foenum-graecum* should be further studied for its natural bioactive compounds, which have great therapeutic potential.

## Figures and Tables

**Table 1:** The physicochemical properties of fenugreek seed oil.

Acid Value Mg KOH/g	Saponification Value Mg KOH/g	Ester Value	Free Fatty Acids mg/100 g Oil	Refractive Index
4.75	195	190.25	2.38	1.4640

**Table 2:** The phytochemical screening test for Fenugreek seeds.

Main Class	Status
Alkaloids	+
Tannins	+
Saponins	+
Glycosides	+
Flavonoids	+
Steroids	+
Terpenoids	−

− Means the main class was not detected. + Means the main class was detected.

**Table 3:** GC-MS analysis of ethanol extract of fenugreek seed.

No.	R. Time	Compound Name	Mol. Form	% Area
**1**	5.12	Nitrobutan-3-ol	C_4_H_9_NO_3_	**0.61**
**2**	5.54	Dimethyl trisulfide	C_2_H_6_S_3_	4.23
**3**	6.04	Ethylmethane Sulfonate	C_3_H_8_O_3_S	12.41
**4**	6.62	N-methylhomopiperazine	C_6_H_14_N_2_	3.22
**5**	7.12	Methylsulfonyl methane	C_2_H_6_O_4_S	1.24
**6**	8.24	2-Furanmethanol	C_5_H_6_O_2_	5.32
**7**	9.33	Trigonelline	C_7_H_7_NO_2_	3.21
**8**	12.43	β-Estradiol-3-methyl ether	C_19_H_26_O_2_	2.33
**9**	14.2	Cyclohexasiloxane dodecamethyl-4,4-methyl-	C_12_H_36_O_6_Si_6_	2.31
**10**	15.13	Decanoic acid	C_10_H_20_O_2_	0.74
**11**	15.62	4-Hydroxybenzoic acid	C_7_H_6_O_3_	2.45
**12**	18.03	4-Methylaminobenzoic acid	C_8_H_9_NO_2_	4.21
**13**	18.76	2-Methylundecanal	C_12_H_24_O	2.15
**14**	19.28	Didodecyl phthalate	C_32_H_54_O_4_	0.89
**15**	19.88	4-Butyl-2(4-nitrophenyl)-1,3-thiazole	C_13_H_14_N_2_O_3_S	8.21
**16**	20.21	n-Hexadecanoic acid	C_16_H_32_O_2_	9.12
**17**	20.74	Apigenin 6,8-di C-glucoside	C_27_H_30_O_15_	0.83
**18**	21.35	1,4-Benzenedicarboxylic acid dimethyl ester	C_10_H_10_O_4_	3.86
**19**	21.94	Pentanal	C_5_H_10_O	3.45
**20**	22.54	9,12-Octadecadienoic acid (Z,Z)-	C_18_H_32_O_2_	6.12
**21**	23.13	Diosgenin	C_27_H_42_O_3_	3.65
**22**	23.61	9-Octadecatrienoic acid	C_18_H_34_O_2_	6.23
**23**	24.11	α-Tocopherol	C_29_H_50_O_2_	5.46
**24**	24.65	Qurecetin 3-arabinoside	C_20_H_18_O_11_	3.24
**25**	24.91	Cholest-5-ene 3-bromo-3β-	C_27_H_45_Br	4.51
				100

**Table 4:** The inhibition zone of Fenugreek hydroethanolic extract expressed as means of three replicates (mean ± SD).

Microorganisms Tested	Fenugreek Extract			Standard Antimicrobial *
	1 mg/disc	2 mg/disc	3 mg/disc	
***S. aureus***	9.12 ± 1.15 ^c^*	12.84 ± 0.52 ^b^	15.02 ± 0.50 ^c^	25.25 ± 1.12 ^a^
***E. coli***	12.56 ± 0.12 ^b^	13.44 ± 0.25 ^b^	16.11 ± 0.10 ^b^	11.22 ± 0.33 ^c^
***Sal. Typhyimurium***	8.72 ± 0.30 ^c^	13.00 ± 0.30 ^b^	15.24 ± 0.10 ^c^	7.42 ± 0.27 ^d^
***A. flavus***	14.11 ± 0.12 ^a^	15.30 ± 0.32 ^a^	16.87 ± 0.32 ^a^	21.65 ± 1.22 ^b^

Different letters reflected different significant levels in respect to mean ± SD; *: Ampicillin for bacterial strains and amphotericin B for *A. flavus*.3.5. In Silico/Computational Modeling of Fenugreek.

**Table 5:** Binding energy of the identified compound and the 3 targeted receptors: 1JIJ, 2QZW and 1HD2 for TyrRS from *Staphylococcus aureus*, aspartic proteinase from *Candia albicans*, and Human Peroxiredoxin 5 (PRDX5), respectively.

Receptor/Ligand	Binding Energy (kcal/mol)		
	1JIJ	2QZW	1H2D
1	–5.2	–4.0	–4.2
2	–2.7	–2.3	–2.5
3	–5.1	–4.0	–4.2
4	–4.9	–4.1	–4.3
5	–3.7	–2.9	–3.1
6	–4.8	–4.0	–3.9
7	–6.4	–4.7	–4.7
8	–6.0	–4.8	–5.3
9	–4.9	–5.9	–5.2
10	–5.2	–4.7	–4.2
11	–6.4	–5.0	–5.2
12	–6.5	–4.9	–4.7
13	–5.0	–4.5	–4.1
14	–5.6	–5.7	–4.8
15	–7.5	–6.9	–6.0
16	–5.2	–5.1	–4.2
17	–5.8	–5.5	–4.9
18	–7.1	–5.1	–5.4
19	–4.0	–3.5	–3.4
20	–5.1	–5.5	–4.6
21	–9.4	–8.6	–7.8
22	–5.5	–5.3	–5.0
23	–6.4	–6.7	–6.1
24	–8.6	–8.0	–8.3
25	–6.0	–5.5	–5.8

**Table 6:** Interactions, bond category, and closest interacting residues for the best-identified compounds with the targeted receptors: 1JIJ, 2QZW, and 1H2D for TyrRS from *Staphylococcus aureus*, aspartic proteinase from *Candia albicans*, and human peroxiredoxin 5 (PRDX5) respectively.

Compound No.	No. H-Bond	Interacting Residues	
		Closest Interacting Residues (Distance, Å)	No. Closest Interacting Residues (Å)
TyrRS from *Staphylococcus aureus* (1JIJ)			
**21**	2	**Carbon H-bond:** Gly49 (3.53), Gly238 (3.54); **Alkyl:** Leu223 (5.26), Val224 (5.28), Lys234 (4.90), Lys234 (4.09), Ala239 (4.92), Leu52 (4.47), Pro53 (5.05), Val224 (4.24)	8
**24**	7	**Conventional H-bond:** Arg58 (2.01), Arg58 (2.28), Lys305 (2.13), Asn109 (2.51), Glu302 (2.87), Glu112 (2.95), Glu62 (2.20); **π-Anion** Glu62 (4.12); **π-π Stacked:** Phe306 (4.33)	7
**15**	4	**Attractive charge:** Lys84 (3.94), Arg88 (5.25); **Conventional H-bond:** Lys84 (2.79), Lys84 (2.71), Asn124 (2.90), Gly38 (2.47); **Alkyl:** Ala39 (4.55), Pro53 (4.37); **π-Alkyl:** His50 (4.44), His50 (4.17), Phe54 (4.93)	8
Aspartic proteinase from *Candia albicans* (2QZW)			
**21**	2	**Conventional H-bond:** Ser12 (2.78); **Carbon H-bond:** Lys49 (3.54); **Alkyl:** Val12 (4.79), Pro120 (3.90), Pro120 (4.33)	4
**24**	7	**Conventional H-bond:** Arg192 (2.69), Arg195 (2.31), Arg195 (2.46), Asp32 (2.29), Asp218 (2.56), Thr221 (2.35), Asp37 (2.77); **Carbon H-bond** Glu193 (3.24); **π-π T-shaped:** Tyr84 (5.42); **π-Alkyl:** Ala133 (5.47)	9
**15**	4	**Conventional H-bond:** Gly85 (1.88), Thr222 (2.46), Ile223 (2.73), Gly220 (2.86); **π-Anion:** Asp86 (4.95), Asp86 (3.78), Asp218 (4.44); **Alkyl:** Ile30 (4.09)	7
Human peroxiredoxin (PRDX5, 1HD2)			
**24**	6	**Conventional H-bond:** Asn21 (2.48), Arg86 (2.45), Arg86 (2.27), Gly82 (2.03); **π-Anion:** Glu16 (4.28); **π-Donor H-bond:** Gly92 (2.69); **π-Sigma:** Leu96 (3.99); **Amide-π Stacked:** Gly92:C,O;Lys93:N (4.34); **π-Alkyl:** Arg95 (5.12), Arg95 (4.77), Ala90 (4.84)	9
**21**	1	**Conventional H-bond:** Arg124 (2.78); **Alkyl:** Pro45 (5.08), Ile119 (4.91); **π-Alkyl:** Phe120 (5.26)	4
**23**	2	**Conventional H-bond:** Arg86 (2.33), Arg86 (2.35); **π-Anion:** Glu16 (3.33); **Alkyl:** Val69 (5.12), Arg95 (4.33), Lys32 (4.16), Val69 (3.74)	5

**Table 7:** Lipophilicity, pharmacokinetics, drug-likeness and medicinal chemistry of the identified compounds based on the ADME/Tox (for absorption, distribution, metabolism, excretion and toxicity) properties.

Entry	1	2	3	4	5	6	7	10	11	12	13	15	16	18	19	20	22	24
	Lipophilicity & Physicochemical Properties																	
TPSA	66.05	75.9	51.75	15.27	42.52	33.37	44.01	37.3	57.53	49.33	17.07	127.08	37.3	74.6	17.07	37.3	37.3	227.58
Log Po/w (iLOGP)	1.09	1.91	1.91	1.8	0.6	1.49	−3.11	2.5	0.85	1.17	3.16	2.28	3.85	0.74	1.52	1.52	3.39	1.13
Consensus Log Po/w	−0.24	1.51	1.12	0.32	0.01	0.62	−0.61	3	1.05	1.13	3.87	2.52	5.2	1.13	1.23	1.23	5.07	−0.47
Log S (ESOL) solubility	−0.56	−1.34	−1.09	−0.3	−0.17	−1.09	−1.39	−2.96	−2.07	−2.4	−3.49	−4.27	−5.02	−2.37	−0.86	−0.86	−4.7	−3.27
	Pharmacokinetics																	
GI absorption	High	High	High	Low	High	High	High	High	High	High	High	High	High	High	High	High	High	Low
BBB permeant	No	Yes	Yes	No	Yes	Yes	No	Yes	Yes	Yes	Yes	No	Yes	No	Yes	Yes	Yes	No
P-gp substrate	No	No	No	No	No	No	No	No	No	No	No	No	No	No	No	No	No	Yes
CYP1A2	No	No	No	No	No	No	No	No	No	No	No	Yes	Yes	No	No	No	Yes	No
CYP2C19	No	No	No	No	No	No	No	No	No	No	No	Yes	No	No	No	No	No	No
CYP2C9	No	No	No	No	No	No	No	No	No	No	No	Yes	Yes	No	No	No	Yes	No
CYP2D6	No	No	No	No	No	No	No	No	No	No	No	No	No	No	No	No	No	No
CYP3A4	No	No	No	No	No	No	No	No	No	No	No	No	No	No	No	No	No	No
Log Kp (skin permeation)	High	High	High	Low	High	High	High	High	High	High	High	High	High	High	High	High	High	Low
	Druglikeness & Medicinal chemistry																	
Lipinski	Yes	Yes	Yes	Yes	Yes	Yes	Yes	Yes	Yes	Yes	Yes	Yes	Yes	Yes	Yes	Yes	Yes	No
Biovailability score	0.55	0.55	0.55	0.55	0.55	0.55	0.55	0.85	0.85	0.85	0.55	0.56	0.85	0.85	0.55	0.55	0.85	0.11
Leadlikeness	1	1	1	1	1	1	1	3	1	1	3	1	2	1	1	1	2	1
Synthetic accessibility	2.48	3.64	2.79	1.52	1.76	1.97	1.04	1.67	1	1	2.28	3.07	2.31	1	1	1	3.68	5.26

TPSA: Topological polar surface area; GI: Gastro-intestinal; BBB: Blood-brain-barrier; P-gp: P-glycoprotein; CYP: Cytochrome P450.

## Data Availability

Data are contained within the article.
